# Retrospective analysis of the clinical value of targeted nursing using a game-based model in children undergoing auricular reconstruction for congenital microtia

**DOI:** 10.3389/fped.2026.1826110

**Published:** 2026-07-30

**Authors:** Xue-Zhen Cao, Jun-Qing Wang, Xue-Song Guo

**Affiliations:** Department of Plastic and Burn Surgery, Shanxi Provincial Children’s Hospital, Taiyuan, China

**Keywords:** auricular reconstruction, congenital microtia, game-based model, quality of life, retrospective observational study, self-concept

## Abstract

**Objective:**

To retrospectively evaluate the effects of targeted nursing centered on a game-based model on self-concept, anxiety, and quality of life in children undergoing auricular reconstruction for congenital microtia.

**Methods:**

This retrospective observational study included medical records from children with congenital microtia who underwent auricular reconstruction between January 2022 and December 2025. Medical records were categorized into a control group and an intervention group according to the perioperative nursing model documented during hospitalization. The control group received routine nursing care, whereas the intervention group received routine nursing care combined with game-based nursing. Data from the Piers-Harris Children Self-Concept Scale (CSCS), Screen for Child Anxiety-Related Emotional Disorders (SCARED), and Pediatric Quality of Life Inventory (PedsQL 4.0) scores routinely recorded at admission and discharge during hospitalization were retrospectively extracted from the nursing records.

**Results:**

A total of 70 children were included, with 35 in the routine nursing group and 35 in the game-based nursing group. At admission, no statistically significant differences were found between the two groups in CSCS, SCARED, or PedsQL 4.0 scores (*p* > 0.05). At discharge, the intervention group had higher CSCS scores (69.28 ± 6.75) than the control group (66.19 ± 4.07) (*p* = 0.007), lower SCARED scores (40.56 ± 2.32) than the control group (54.19 ± 2.12) (*p* < 0.001), and higher PedsQL 4.0 scores (70.28 ± 3.16) than the control group (67.19 ± 3.42) (*p* < 0.001).

**Conclusion:**

This retrospective analysis suggests that targeted nursing using a game-based model may be associated with improved perioperative psychological outcomes, particularly reduced anxiety, in children undergoing auricular reconstruction for congenital microtia.

## 1 Introduction

Congenital microtia is a congenital craniofacial anomaly characterized primarily by underdevelopment of the external ear (1). The global incidence rate is 1 to 10 cases per 10,000 live births. Microtia is often associated with external auditory canal atresia and is usually unilateral, with a higher prevalence on the right side. Children with microtia may present with auricular dysplasia accompanied by conductive hearing loss. The primary goals of functional auricular reconstruction are to restore auricular morphology and improve auditory function (2). However, because of limited cognitive and emotional maturity, children may experience heightened anxiety and fear in unfamiliar medical settings. These emotional responses may manifest as resistance to treatment, agitation, or persistent crying, thereby interfering with both clinical management and nursing care procedures (3).

Previous studies have shown that game-based interventions can facilitate emotional regulation in pediatric populations by promoting relaxation, enhancing familiarity with the clinical environment, supporting accurate expression of internal states, correcting maladaptive cognitive patterns, and improving self-concept. These benefits may contribute to better physical and psychological well-being, improved treatment cooperation, and more favorable therapeutic outcomes (4, 5).

Therefore, this study used a retrospective observational design to systematically analyze the effects of game-based nursing on self-concept, anxiety, and quality of life in children undergoing auricular reconstruction for congenital microtia, with the aim of providing evidence for high-quality pediatric nursing care.

## 2 Materials and methods

### 2.1 Study participants

This retrospective observational study included medical records from children with congenital microtia who underwent auricular reconstruction at our hospital between January 2022 and December 2025. According to the perioperative nursing model documented in the medical records, children admitted from January 2022 to June 2024 were categorized as the control group, which received routine nursing care, whereas children admitted from July 2024 to December 2025 were categorized as the intervention group, which received routine nursing care combined with game-based nursing. Thus, the study used a retrospective historical before-and-after comparison based on two consecutive admission periods.

Inclusion criteria were as follows: (1) diagnosis of congenital microtia and receipt of auricular reconstruction; (2) age 5–12 years; (3) completion of scale assessments during hospitalization; and (4) complete clinical medical records. Exclusion criteria were as follows: (1) more than two previous auricular reconstruction procedures; (2) organic diseases of the heart, liver, kidneys, or other organs; (3) cognitive, language, or hearing impairments; (4) psychiatric disorders or a family history of psychiatric illness.

As this was a retrospective observational study, the sample size was determined by the number of eligible patients with complete medical records during the study period. No formal *a priori* sample size calculation was performed.

### 2.2 Methods

The control group comprised patients admitted from January 2022 to June 2024 who received routine perioperative nursing care throughout hospitalization. Routine care included conventional health education on the day before surgery, preoperative preparation on the morning of surgery, and postoperative symptomatic nursing care as prescribed by physicians. No specialized psychological intervention was provided, and nursing procedures followed the department's existing nursing schedule.

The intervention group comprised children admitted from July 2024 to December 2025. In addition to the routine nursing care provided to the control group, these children received a game-based nursing intervention delivered at standardized time points according to the following structured timeline (6):

(1) Day of admission (2–4 h after admission): initial psychological assessment and environmental adaptation guidance;
(2) One day before surgery (16:00–17:00): surgical education, game-based counseling, and family-coordinated education;
(3) 30 min before surgery: bedside emotional soothing and relaxation guidance;
(4) 6 h after surgery and on postoperative days 1 and 3 at fixed times: game-based interventions were conducted.

All interventions were delivered by designated specialized nurses at the standardized time points. Quality control nurses verified the implementation time of each intervention against nursing execution records to ensure uniform intervention timing, process traceability, and consistency of delivery (7).

To ensure intervention fidelity and consistency, a standardized perioperative nursing protocol was developed. All participating nurses received specialized training and passed competency assessments before the study. The intervention content, health education standards, activity frequency, and implementation timing were uniformly defined. A dedicated quality control nurse supervised the entire process to minimize performance bias and ensure consistent delivery of the intervention.

### 2.3 Outcome measures

Archived scale assessment data were retrieved. Outcome assessors were aware of group allocation, and blinded outcome assessment was not implemented.

#### 2.3.1 Piers–harris children self-concept scale (CSCS)

The CSCS was used to assess self-concept in pediatric participants. Self-concept encompasses an individual's perceptions, evaluations, and attitudes toward their behavior, capabilities, and values, reflecting their awareness of their position within the environment and society (8). A diminished self-concept is associated with reduced self-esteem and confidence and may manifest as feelings of inferiority, self-neglect, or social withdrawal, requiring timely intervention. The CSCS consists of 80 items (yes = 1, no = 0), with total scores ranging from 0 to 80. Higher scores indicate higher levels of self-concept. The scale demonstrates strong internal consistency, with a Cronbach's *α* coefficient of 0.821. The CSCS is generally recommended for children aged 6–16 years. In this study, two participants aged 5 years were assessed by their parents based on the children's daily performance, while children aged 6–12 years completed the scale independently.

#### 2.3.2 Screen for child anxiety-related emotional disorders (SCARED)

The SCARED is a validated tool designed to screen for anxiety-related symptoms in pediatric populations. Originally developed by Birmaher et al. (9) in 1997 and revised in 1999, the instrument comprises 41 items, each rated on a 3-point Likert scale: 0 (not true or hardly ever true), 1 (somewhat true or sometimes true), and 2 (very true or often true). Total scores range from 0 to 82. The instrument assesses 5 domains of anxiety: somatization/panic disorder, generalized anxiety disorder, separation anxiety disorder, social phobia, and school phobia (10). Higher scores indicate greater severity of anxiety symptoms. The SCARED is applicable to children aged 8–18 years. In this study, children aged 5–7 years were assessed by their accompanying parents using the parent rating scale based on daily performance, while children aged 8–12 years completed the child self-report scale independently.

#### 2.3.3 Pediatric quality of life inventory (PedsQL 4.0)

The PedsQL 4.0 was employed to evaluate health-related quality of life. It includes 23 items assessing physical, emotional, social, psychological, and school functioning (11). Two versions are available, child self-report and parent proxy-report. Items are rated on a 5-point Likert scale (0 = never a problem to 4 = almost always a problem), with a total scores ranging from 0 to 92. Higher scores indicate better quality of life. The PedsQL 4.0 is applicable for children aged 2–18 years. Children aged 5–7 years used the young-child version (ages 5–7) with the parent-proxy scale completed simultaneously. Children aged 8–12 years used the school-age version, accompanied by the parent-proxy questionnaire.

### 2.4 Statistical analysis

All statistical analyses were performed using SPSS version 26.0 (IBM Corp., Armonk, NY, USA). Continuous variables were expressed as mean ± standard deviation (SD). Between-group comparisons were performed using independent-samples *t*-tests, while within-group comparisons were conducted using paired-samples *t*-tests. A *p* value of <0.05 was considered statistically significant. Effect sizes (Cohen's d) were interpreted as follows: *d* < 0.2 = negligible, 0.2 ≤ d < 0.5 = small, 0.5 ≤ *d* < 0.8 = moderate, and *d* ≥ 0.8 = large.

## 3 Results

### 3.1 Baseline characteristics

A total of 70 eligible children were included in the final analysis, including 35 in the control group and 35 in the intervention group. In the intervention group, there were 23 males and 12 females; the affected side was left in 13 cases (37.14%) and right in 22 cases (62.85%); the age range was 5–12 years, with a mean age of 7.3 ± 1.7 years. In the control group, there were 24 males and 11 females; the affected side was left in 15 cases (42.86%) and right in 20 cases (57.14%); the age range was 6–12 years, with a mean age of 8.0 ± 1.9 years. Baseline clinical characteristics were comparable between the two groups, with no statistically significant differences (*p* > 0.05).

### 3.2 Scale scores at admission

At admission, there were no statistically significant differences in CSCS, SCARED, or PedsQL 4.0 scores between the two groups (*p* > 0.05), indicating comparable baseline status.

### 3.3 Comparison of scale scores at discharge

#### 3.3.1 CSCS scores

At discharge, the intervention group had significantly higher CSCS and PedsQL 4.0 scores and significantly lower SCARED scores than the control group (*p* < 0.05).

At admission, there was no significant difference in CSCS scores between the two groups (*p* = 0.431), indicating comparable baseline self-concept. At discharge, CSCS scores in both groups were significantly higher than those at admission (both *p* < 0.001). The intervention group showed a greater increase in CSCS scores and had significantly higher discharge scores than the control group (69.28 ± 6.75 vs. 66.19 ± 4.07, *p* = 0.007), suggesting that the game-based nursing model had a more pronounced effect on improving self-concept in children, as shown in Table 1. The mean difference between groups was 3.09 points (95% CI: 0.48, 5.70). Cohen's d was 0.55, indicating a moderate effect.

**Table 1:** Comparison of CSCS scores between the two groups (mean ± SD).

Group	*n*	At admission ($\bar{x}\pm s$)	At discharge ($\bar{x}\pm s$)	*T*	*p*-value
Intervention group	35	54.18 ± 1.05	69.28 ± 6.75	2.772	<0.007
Control group	35	53.89 ± 2.37	66.19 ± 4.07		
*t*		0.791			
*P*		0.431			

#### 3.3.2 SCARED scores

At discharge, SCARED scores in both groups were significantly reduced (both *p* < 0.001). The reduction in anxiety was significantly greater in the intervention group than in the control group (40.56 ± 2.32 vs. 54.19 ± 2.12, *p* < 0.001), indicating that the game-based nursing model was more effective in alleviating anxiety-related symptoms in children (Table 2). The mean difference between groups was −13.63 points (95% CI: −14.67, −12.59). Cohen's d was 6.05, indicating a very large effect.

**Table 2:** Comparison of SCARED scores between the two groups (mean ± SD).

Group	*n*	At admission ($\bar{x}\pm s$)	At discharge ($\bar{x}\pm s$)	*T*	*p*-value
Intervention group	35	62.67 ± 3.04	40.56 ± 2.32	30.677	<0.001
Control group	35	63.64 ± 3.52	54.19 ± 2.12		
*t*		1.475			
*P*		0.143			

#### 3.3.3 PedsQL 4.0 scores

At admission, there was no statistically significant difference in PedsQL 4.0 scores between the two groups (*p* = 0.320). At discharge, quality-of-life scores improved significantly in both groups (both *p* < 0.001); the improvement was more pronounced in the intervention group, whose discharge scores were significantly higher than those of the control group (70.28 ± 3.16 vs. 67.19 ± 3.42, *p* < 0.001). These findings indicate that implementation of the game-based nursing model was associated with higher PedsQL 4.0 scores at discharge in this population (Table 3). The mean difference between groups was 3.09 points (95% CI: 1.55, 4.63). Cohen's d was 0.94, indicating a large effect.

**Table 3:** Comparison of PedsQL 4.0 scores between the two groups (mean ± SD) .

Group	*n*	At admission ($\bar{x}\pm s$)	At discharge ($\bar{x}\pm s$)	*T*	*p*-value
Intervention group	35	53.23 ± 2.65	70.28 ± 3.16	4.692	<0.001
Control group	35	53.72 ± 2.23	67.19 ± 3.42		
*t*		1.000			
*P*		0.320			

## 4 Discussion

Congenital microtia is a common congenital craniofacial developmental abnormality. Autologous rib cartilage auricular reconstruction is currently the mainstream surgical technique for correcting auricular defects and reconstructing the anatomical shape of the external ear, with mature clinical application and established efficacy. However, this procedure involves substantial surgical trauma and a prolonged postoperative shaping period. Because most affected children are of preschool or school age, they may have limited psychological tolerance and treatment compliance. During the perioperative period, they are prone to anxiety, fear, and agitation, which may affect the shaping outcome of the reconstructed auricle and the postoperative rehabilitation process (12). Therefore, constructing a nursing model that aligns with children's physical and psychological characteristics and meets the specialized needs of auricular reconstruction is a key priority for improving perioperative nursing quality in pediatric plastic surgery.

Conventional routine nursing focuses mainly on disease diagnosis and treatment, standardized condition monitoring, basic care, and health guidance. Such interventions are often monotonous and lack child-friendly elements, making it difficult to effectively alleviate perioperative negative emotions in children undergoing surgery (13). Because children are naturally unfamiliar with and afraid of medical procedures, simple oral education and passive nursing interventions may be insufficient to reduce their resistance to medical care, creating a bottleneck in overall nursing effectiveness (14).

The targeted nursing program in this study centered on a game-based model and integrated situational simulation games, cognitive challenges, attention diversion, and positive reinforcement throughout preoperative education, intraoperative cooperation, postoperative immobilization, and rehabilitation nursing. This approach provided individualized, child-friendly, and specialized nursing interventions. The results showed that the intervention group had significantly lower perioperative anxiety than the control group, which is consistent with previous studies. Xiao et al. (15) confirmed that game-based interventions can reduce the intrusiveness and fear associated with medical procedures through situational simulation, helping children become familiar with the treatment process in advance, reducing anxiety caused by uncertainty at the cognitive level, and improving active cooperation.

The game-based model is a systematic and engaging form of psychological nursing therapy that helps children address psychological problems arising from illness (16). This model proposes that games are a primary means of self-expression for children aged 5–12 years. Through games, children can freely express their emotions, experiences, and inner world (17). Game activities can shorten the psychological distance between nurses and children, promote emotional expression and cognitive development, and help resolve psychological issues (18). In this study, the psychological status and quality of life of the two groups were comparable at admission. Analysis of discharge data showed that children receiving the game-based model had higher self-concept scores, reduced anxiety, and better overall quality of life than those receiving routine nursing. Tan et al. (19) reported that targeted nursing through various game activities can effectively promote children's cognitive development. The congenital microtia knowledge contests used in this study may exercise children's cognitive abilities and disease-related problem-solving skills (20); role-playing based on cartoon characters may help gain children's trust and develop social skills; and diverse recreational activities may contribute to cognitive development, thereby enhancing self-concept (21, 22). The SCARED score was significantly lower in the intervention group than in the control group (40.56 ± 2.32 vs. 54.19 ± 2.12, *P* < 0.05). Wu et al. (23) suggested that different game activities can help identify children's psychological problems and assist them in finding solutions. The PedsQL 4.0 score was significantly higher in the intervention group than in the control group (70.28 ± 3.16 vs. 67.19 ± 3.42, *P* < 0.05). The game-based model also involved parent-child interaction. Through shared activities such as picture book reading and drawing, parent-child interactions promoted communication between children and family members, enabling parents to better understand their child's inner world and establish closer bonds (24). These interactions may help parents master effective communication methods, improve family harmony, and promote children's cognitive, emotional, social, and motor development, thereby enhancing quality of life (25).

Although all three outcome measures showed statistically significant between-group differences, the magnitude and clinical relevance of these differences varied across scales. At discharge, the intervention group had a 13.63-point lower SCARED score than the control group, suggesting a potentially meaningful reduction in perioperative anxiety. In contrast, the between-group differences in CSCS and PedsQL 4.0 scores were approximately 3 points each. For PedsQL 4.0, previously reported minimal clinically important differences are often around 4–7 points for parent-proxy reports; therefore, the observed difference in this study may be considered borderline or modest in clinical magnitude. For CSCS, established minimal clinically important difference data are limited, making the clinical interpretation of a 3-point change less certain. These findings suggest that the game-based nursing model may have its most pronounced short-term effect on reducing perioperative anxiety, whereas its effects on self-concept and quality of life should be interpreted more cautiously.

This study compared the application effects of two nursing intervention models in children aged 5–12 years and provided additional clinical data on psychological status, anxiety, and quality of life in this patient population. The findings supplement domestic retrospective evidence on perioperative psychological nursing for children with congenital microtia and may help strengthen the evidence base for specialized pediatric nursing.

Implications for clinical pediatric nursing practice are as follows:

1. Optimization of nursing protocols: In clinical pediatric nursing, psychological screening of school-age children should be emphasized in addition to routine disease-specific care. Anxiety assessment, self-concept evaluation, and quality-of-life monitoring may be incorporated into routine nursing assessment processes to identify children at high risk of negative emotions as early as possible.
2. Individualized stratified nursing: Given the developmental differences among children aged 5–12 years, differentiated nursing should be implemented according to age. For younger children aged 5–7 years, parent-coordinated nursing and game-based guidance should be the main approaches. For children aged 8 years and above, communication education and social support interventions should be emphasized to achieve stratified and refined nursing.
3. Nursing quality control: Psychological outcome indicators may be incorporated into pediatric nursing quality-control systems. Physical rehabilitation should not be the sole criterion for evaluating treatment efficacy. This shift may promote the transformation of pediatric nursing from a single physical care model to an integrated biopsychosocial nursing model.

Several limitations should be acknowledged. First, this was a single-center, non-randomized, historical before-after observational study based on admission period, which may have introduced selection bias and time-related bias. Second, no formal *a priori* sample size calculation was performed, and the sample size was limited; therefore, the study may have been underpowered to detect smaller but potentially meaningful effects, particularly for CSCS and PedsQL 4.0. Third, multiple primary outcomes were assessed without adjustment for multiple comparisons, which may increase the risk of type I error. Fourth, the use of parametric t-tests was based on the available retrospective dataset, and additional distributional diagnostics could not be performed retrospectively; therefore, the robustness of these comparisons should be interpreted cautiously. Fifth, outcomes were assessed using subjective scales, parent-proxy versions were used for younger children aged 5–7 years, outcome assessors were aware of group allocation, and blinded outcome assessment was not implemented; therefore, assessment bias cannot be excluded. Finally, no long-term follow-up was conducted, limiting evaluation of the durability of improvements in self-concept and quality of life. Future multicenter, prospective randomized controlled studies with larger samples, predefined sample size estimation, blinded outcome assessment, correction for multiple outcomes, and extended follow-up are needed to further validate the clinical effectiveness of game-based nursing in this population.

## 5 Conclusion

In conclusion, this retrospective study suggests that targeted nursing centered on a game-based model may help reduce perioperative anxiety in children undergoing auricular reconstruction for congenital microtia. Its effects on self-concept and quality of life appear favorable but should be interpreted cautiously given the modest between-group differences and the retrospective design.

## Data Availability

The original contributions presented in the study are included in the article/Supplementary Material, further inquiries can be directed to the corresponding author/s.

## References

[1] Brandt HH Bodmer D. Aktuelle Diagnostik und Therapie bei Ohrmuscheldysplasien und gehörgangsfehlbildungen. HNO. (2024) 72(1):57–68. 10.1007/s00106-023-01386-8 38047932 PMC10781867

[2] Zhang M Lin J Zhang J Han Z Liu C Zou Y. A clinical epidemiological study on congenital ear malformation (CEM). Acta Otolaryngol. (2023) 143(sup1):S17–24. 10.1080/00016489.2023.2276348 38071650

[3] Ku PKM Vlantis AC Tong MC Chan TTT Yeung ZWC Cho RHW. A hybrid auricular framework of autologous rib cartilage and a porous polyethylene implant for reconstruction of congenital microtia: a modification of nagata’s technique. Facial Plast Surg Aesthet Med. (2024) 26(1):15–22. 10.1089/fpsam.2022.0152 37256708 PMC10794839

[4] Sun P Wang C Huang X Pan B. A novel method to accurately locate the reconstructed auricle. Transl Pediatr. (2022) 11(4):487–94. 10.21037/tp-21-453 35558970 PMC9085952

[5] Wang Y Liu Y Zhu J Yang J Wang D Zhao S. Analysis of predictive factors related to long-term hearing and temporal bone development in congenital microtia. Int J Pediatr Otorhinolaryngol. (2024) 176:111838. 10.1016/j.ijporl.2023.111838 38168652

[6] Liao RR Lan P Kang J Chen YP Wang Q Huang MY. Influence of medical game counseling on psychological status and pain in school-age children with microtia. Chin Mod Med. (2025) 32(20):147–51. 10.3969/j.issn.1674-4721.2025.20.32

[7] Li L Yan H Peng XY. Effects of specialized game intervention on negative emotions and stress response in children with congenital microtia undergoing auricular reconstruction. Chin Gen Pract Nurs. (2022) 20(16):2230–3. 10.12104/j.issn.1674-4748.2022.16.020

[8] Wang Y Jiang H Pan B Ma L Zhou J Song Y. Correlations among clinical phenotypes, radiological examination indexes, and hearing Status in congenital microtia. J Craniofac Surg. (2024) 35(3):860–4. 10.1097/SCS.0000000000009867 38011621

[9] Birmaher B Khetarpal S Brent D Cully M Balach L Kaufman J. The screen for child anxiety related emotional disorders (SCARED): scale construction and psychometric characteristics. J Am Acad Child Adolesc Psychiatry. (1997) 36(4):545–53. 10.1097/00004583-199704000-00018 9100430

[10] Sun P Lin Q Zhang M Liu Z Zhu L. Epidemiological study of neonatal congenital microtia in shandong province, China, 2011–2020. J Craniofac Surg. (2022) 33(8):e828–31. 10.1097/SCS.0000000000008761 35848724

[11] Qian YZ Wang WT Chen QJ Ning Q. The impact of strabismus correction surgery on the quality of life of children. Nurs Pract Res. (2021) 18(21):3281–4. 10.3969/j.issn.1672-9676.2021.21.031

[12] Liu Y Zhao SJ. The effect of narrative therapy on the improvement of mental flexibility of AdolescentPatients with congenital microtia. Chin Sci J Hear Speech Rehabil. (2020) 18(2):135–8. 10.3969/j.issn.1672-4933.2020.02.014

[13] Wang L Li DY Nong L Yuan W. Research progress on nursing mode of auricular reconstruction surgery for microtia. Chin J Aesthetic Plast Surg. (2024) 35(11):701–4. 10.3969/j.issn.1673-7040.2024.11.018

[14] Gao QQ Pang QH Zhang YY. Analysis of medical fear emotion and its influencing factors in school aged children with congenital heart disease. Chin J Health Psychol. (2025) 33(4):568–72. 10.13342/j.cnki.cjhp.2025.04.018

[15] Xiao M Xiao JM Shen Q. Application of specialized game intervention in perioperative nursing of burn children with skin grafting. Chin J Aesthetic Med. (2024) 33(3):171–4. 10.15909/j.cnki.cn61-1347/r.006200

[16] Zou YH Sun BC Liu C Lin JH Zhang M Duan XH. Innovated surgery incision for patients of congenital malformation of the middle and outer ear with infection (CMMOEI). Acta Otolaryngol. (2023) 143(sup1):S60–3. 10.1080/00016489.2023.2272683 38071656

[17] Kang C Han L Zhao Y Sun P Gao M Ma M. Multislice spiral computer tomography findings of simple congenital middle ear malformations. Contrast Media Mol Imaging. (2022) 2022:7303647. 10.1155/2022/7303647 35992540 PMC9365615

[18] Sun P Gu L Yu Q Luan F. Pathogenic genes for congenital microtia: a bioinformatics analysis. J Craniofac Surg. (2023) 34(8):2560–2. 10.1097/SCS.0000000000009620 37643078

[19] Tan CH Song BH Ma SS Zhao F She YJ Liu DZ. Development and trend of behavioral intervention research methods in children with autism. Chin J Clin Psychol. (2021) 29(2):436–42. 10.16128/j.cnki.1005-3611.2021.02.044

[20] Niu CP. Research on play-based kindergarten curriculum reform in China: based on the analysis of three typical play modes. Int J Early Years Educ. (2023) 31(2):401–18. 10.1080/09669760.2023.2168520

[21] Duan X Wang Q Wang T Sun B Yang S Zou Y. Study of hearing features of congenital malformation of the middle and outer ear (CMMOE). Acta Otolaryngol. (2023) 143(sup1):S25–9. 10.1080/00016489.2023.2271087 38113147

[22] Zhang Q Guo JL Cheng H Huang YB Bai F Xiang YY. Design and application of a perioperative therapeutic play program for school-aged children with fractures based on comfort theory. Chin J Nurs Educ. (2023) 20(03):333–8. 10.3761/j.issn.16725-9234.2023.03.016

[23] Wu XH Zhou LJ Yu J Xu JY Ye MN Sun L. Design and application of psychological preparation play for pre-magnetic resonance imaging examination in preschool children. Chin J Nurs Educ. (2023) 20(3):338–42. 10.3761/j.issn.1672-9234.2023.03.017

[24] Yu X. Research on the Cultivation of Children’s Creativity Through Thematic Ink Wash Graffiti and Chalk Painting. Liaoning: Liaoning Normal University (2023).

[25] Huang N Fu M Gao F Wang Y Lu M Li X. Influence of targeted nursing-guided bladder filling on embryo transfer outcomes and patient comfort: a prospective open randomized controlled study. Technol Health Care. (2024) 32(3):1421–9. 10.3233/THC-230380 38007682

